# LRRK2 G2019S promotes astrocytic inflammation induced by oligomeric α-synuclein through NF-κB pathway

**DOI:** 10.1016/j.isci.2023.108130

**Published:** 2023-10-05

**Authors:** Kai-Jie He, Jin-Bao Zhang, Jun-Yi Liu, Feng-Lun Zhao, Xiao-Yu Yao, Yu-Ting Tang, Jin-Ru Zhang, Xiao-Yu Cheng, Li-Fang Hu, Fen Wang, Chun-Feng Liu

**Affiliations:** 1 Department of Neurology and Clinical Research Center of Neurological Disease, the Second Affiliated Hospital of Soochow University, Suzhou, Jiangsu 215004, China; 2 Jiangsu Key Laboratory of Neuropsychiatric Diseases and Institute of Neuroscience, Soochow University, Suzhou, Jiangsu 215123, China; 3 Department of Neurology, Dushu Lake Hospital Affilicated to Soochow University, Suzhou, Jiangsu 215123, China; 4 Department of Neurology, The Second Affiliated Hospital of Xinjiang Medical University, Urumqi, Xinjiang 830063, China

**Keywords:** Biological sciences, Molecular biology, Neuroscience, Immunology

## Abstract

Parkinson’s disease (PD) is characterized by the irreversible loss of dopaminergic neurons and the accumulation of α-synuclein in Lewy bodies. The oligomeric α-synuclein (O-αS) is the most toxic form of α-synuclein species, and it has been reported to be a robust inflammatory mediator. Mutations in *Leucine-Rich Repeat Kinase 2* (*LRRK2*) are also genetically linked to PD and neuroinflammation. However, how O-αS and LRRK2 interact in glial cells remains unclear. Here, we reported that *LRRK2 G2019S* mutation, which is one of the most frequent causes of familial PD, enhanced the effects of O-αS on astrocytes both *in vivo* and *in vitro*. Meanwhile, inhibition of LRRK2 kinase activity could relieve the inflammatory effects of both *LRRK2 G2019S* and O-αS. We also demonstrated that nuclear factor κB (NF-κB) pathway might be involved in the neuroinflammatory responses. These findings revealed that inhibition of *LRRK2* kinase activity may be a viable strategy for suppressing neuroinflammation in PD.

## Highlights

• *LRRK2 G2019S* and oligomeric α-synuclein activate astrocytic neuroinflammation
• *LRRK2 G2019S* accelerates dopaminergic cell loss induced by oligomeric α-synuclein
• LRRK2 kinase inhibitor reduces NLRP3 activation and the level of inflammatory factors
• *LRRK2 G2019S* participates in neuroinflammation via regulating the NF-κB pathway

## Graphical abstract

**Figure:**
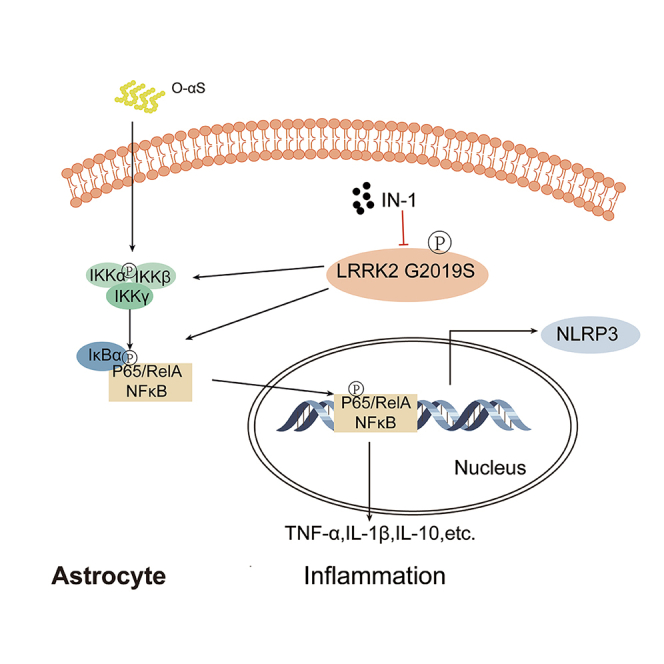

## Introduction

Parkinson’s disease (PD) is the second most frequent neurodegenerative disorder and the fastest growing neurological disease.^1^ PD is mainly characterized by the loss of dopaminergic neurons in the substantia nigra, and the aggregation of misfolded alpha-synuclein (α-syn) to form Lewy bodies.^2^ In recent years, both *in vivo* and *in vitro* evidences have revealed that oligomeric α-syn (O-αS) is the most cytotoxic component leading to neurodegeneration in PD.^3^ A clinical longitudinal study also showed that O-αS in cerebrospinal fluid (CSF) was correlated with the severity of PD motor symptoms.^4^ However, the precise molecular mechanisms of O-αS toxicity remain to be elucidated. Release of pathological O-αS from damaged neurons could accelerate neuronal cell death in part via astrocytes and microglia activation, revealing that neuroinflammation induced by O-αS is an inescapable part of PD pathology.^5^^,^^6^

*Leucine-Rich Repeat Kinase 2* (*LRRK2*) *G2019S* mutation is a common genetic variant in PD patients, accounting for 4% of the familial and 1% of the sporadic PD cases.^2^ Clinical studies have shown that PD patients with *LRRK2 G2019S* mutation have an increase in inflammatory factors compared with patients with primary PD.^7^^,^^8^ Recent study has also revealed higher levels of O-αS and tumor necrosis factor alpha (TNF-α) in the CSF of both symptomatic and asymptomatic *LRRK2* mutation carriers,^9^ indicating that there might be a close interaction between LRRK2 dysfunction and O-αS toxicity in the neuroinflammation associated with PD.

Exploring the interaction of LRRK2 and O-αS could be helpful for developing effective targeted disease-modifying therapies. In the present study, we found that *LRRK2 G2019S* mutation aggravated the glial inflammatory response induced by O-αS, mainly affecting the morphology of astrocytes, and inhibition of LRRK2 kinase activity reduced the production of inflammatory factors induced by O-αS. We also demonstrated that *LRRK2 G2019S* mutation and O-αS might interplay in the nuclear factor κB (NF-κB) pathway, promoting neuroinflammation.

## Results

### *LRRK2 G2019S* induced early parkinsonism-like phenotypes and aggravated the loss of dopaminergic neurons in O-αS-induced mouse model

**Figure 1:**
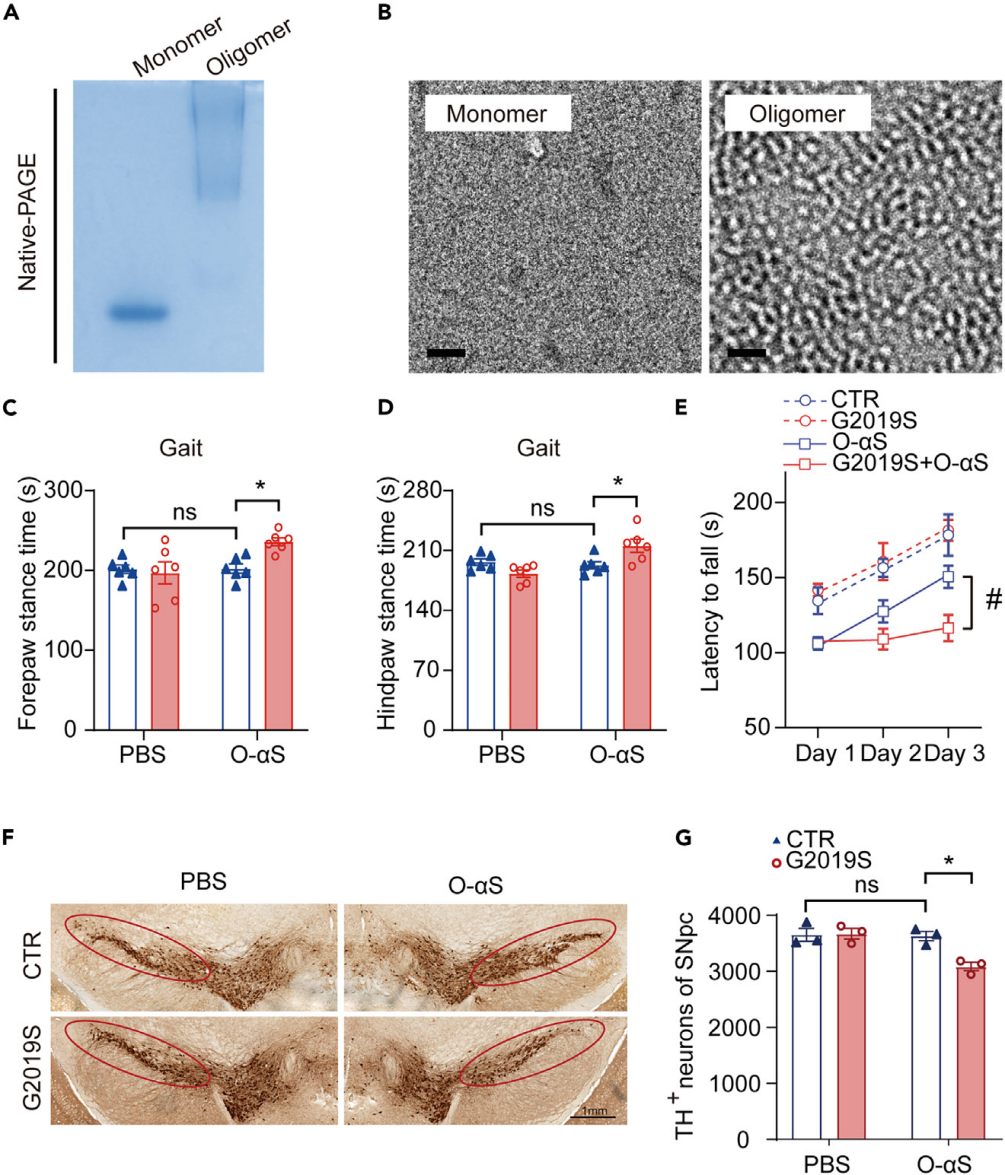
*LRRK2 G2019S* promoted early parkinsonism-like behaviors and dopaminergic neuronal loss in O-αS-treated mouse model (A) Identification of α-syn protein (monomer and oligomer) with Coomassie blue-stained native-PAGE. (B) Representative electron microscopy image of α-syn oligomers (scale bar = 50 nm). (C and D) Stance time of control (CTR), G2019S, O-αS, and G2019S+O-αS mice in gait performance. Two-way ANOVA followed by Turkey’s post-hoc test, n = 6. ∗p < 0.05; ns, not significant. (E) Latency to fall off the accelerating Rota-rod during the days training of the between G2019S group and G2019S+O-αS group. Two-way ANOVA followed by Turkey’s post-hoc test, n = 6. ^#^p < 0.05. (F) Representative images of IHC staining of TH-positive neurons in the substantia nigra pars compacta (SNpc) of mice in the control, G2019S, O-αS, and G2019S+O-αS groups. (scale bar = 1 mm). (G) Statistical comparison of TH-positive neurons of control, G2019S, O-αS, and G2019S+O-αS groups in the substantia nigra pars compacta. Two-way ANOVA followed by Sidak’s post-hoc test, n = 3. ∗p < 0.05; ns, not significant. Data are represented as mean ± SEM.

We first expressed monomeric α-syn in BL21 (DE3) Escherichia coli and purified the protein. Protein purity was confirmed by Coomassie-stained SDS-PAGE and western blotting (Figures S1A and S1B). After removing the endotoxin, we generated a β-sheet-rich oligomer strain (named as type-B∗ oligomer) which had been previously identified to reproduce a variety of pathophysiological effects in neuronal and glial cells.^10^^,^^11^^,^^12^ In native-PAGE, O-αS showed a diffused band with higher molecular mass compared with the monomer (Figure 1A). We also utilized negative staining to further characterize the O-αS. As previously reported, O-αS species appeared to be approximately 5–20 nm in height (Figure 1B).^10^ The soluble O-αS is attributed to specific structural characteristics that confer damaging properties, causing lipid peroxidation and rapidly inducing a large number of intracellular reactive oxygen species (ROS) (Figure S1C).^12^ The β-sheet structure in the prepared O-αS was confirmed using circular dichroism (CD) spectra (Figure S1D).

We stereotactically injected O-αS into the striatum of 6-month-old *LRRK2 G2019S*-Tg mice, and the injected protein was detectable in tyrosine hydroxylase (TH)-positive nigra dopaminergic neurons using anti-human α-syn antibody 7 days post-injection (Figure S1E). To confirm whether O-αS-treated *LRRK2 G2019S*-Tg mice showed early parkinsonism-like behaviors, we evaluated gait performance and motor skill learning which might be affected by early synaptic dysfunction in PD pathogenesis.^13^^,^^14^^,^^15^ Gait performance tests revealed that only G2019S+O-αS group showed increased stance time of both forepaw and hindpaw in the treadmill (Figures 1C and 1D). In the motor learning tests, G2019S+O-αS mice fell off from the accelerating Rota-rod faster compared with G2019S mice after 3 days training (Figure 1E). Repeated measures ANOVA revealed a significant interaction between group and day (Figure 1E). Rota-rod data obtained from each date and trial were presented in Figure S2 and Table S1. To investigate whether *LRRK2 G2019S* mutation affected the survival of dopaminergic neurons after O-αS treatment, we used immunohistochemical staining to quantified TH-positive neurons in O-αS-treated control and *LRRK2 G2019S*-Tg mice. The number of TH-positive cells decreased significantly in the O-αS-treated *LRRK2 G2019S*-Tg mice compared with the O-αS-treated control mice (Figures 1F and 1G). We also quantified the density of TH terminals in striatum. The results were consistent with those of TH-positive cell counting (Figure S3).

### *LRRK2 G2019S* regulated the glial inflammatory response induced by O-αS

**Figure 2:**
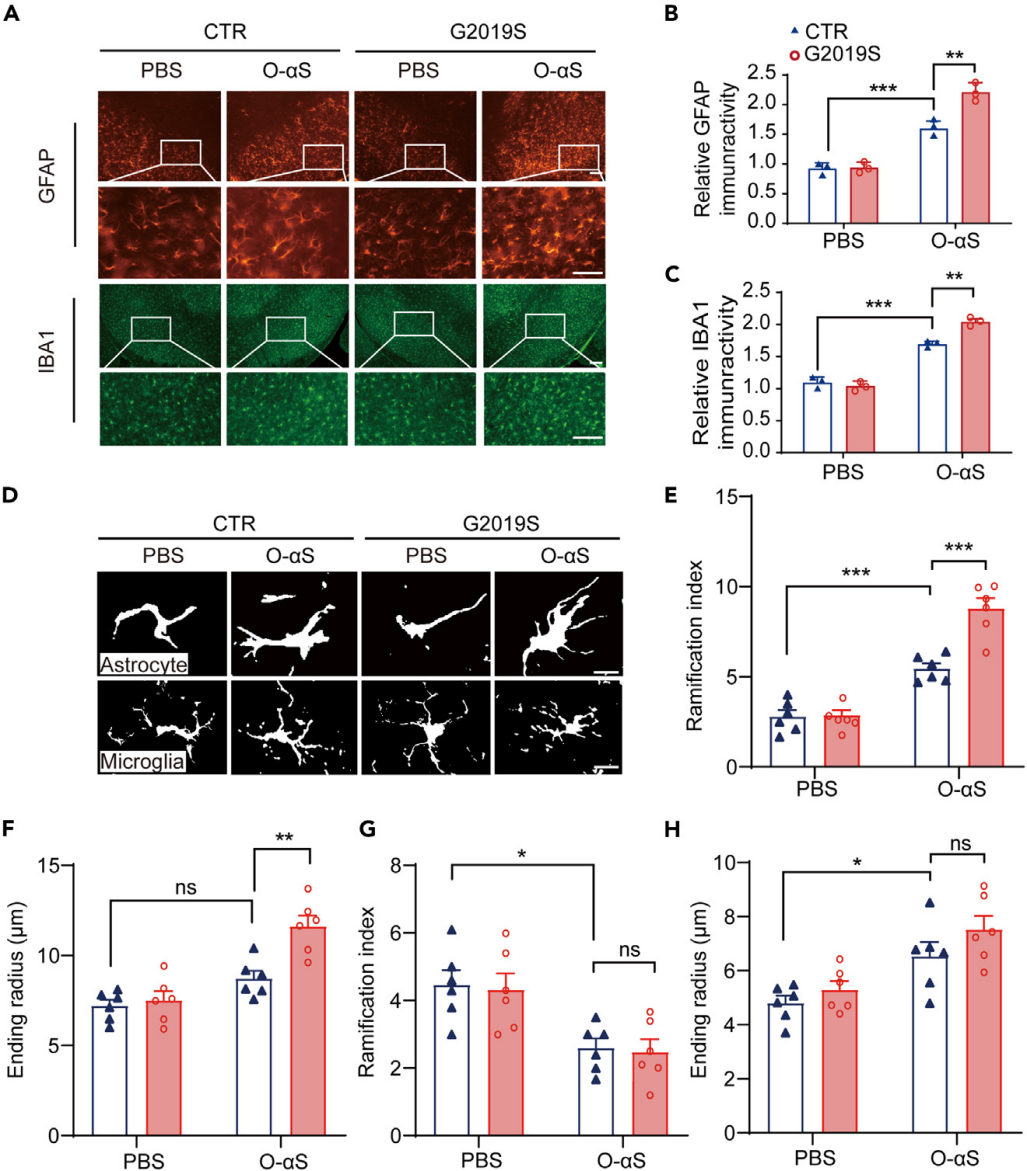
*LRRK2 G2019S* enhanced activation and morphological changes of glia induced by O-αS (A) Immunostaining for astrocyte and microglia on the substantia nigra of mice with anti-IBA1 antibody and anti-GFAP antibody in the control, G2019S, O-αS, and G2019S+O-αS groups. (Scale bar = 50 μm , n = 3 per group). (B) and (C) Statistical comparison of relative fluorescence intensity of control, G2019S, O-αS, and G2019S+O-αS groups reflected activation of astrocytes and microglia. Two-way ANOVA followed by Sidak’s post-hoc test, n = 3. ∗∗p < 0.01; ∗∗∗p < 0.001. (D) Representative images of Sholl analysis for detecting glial morphology. (Scale bar = 10 μm). (E) Morphological (branching index) changes of astrocytes in control, G2019S, O-αS, and G2019S+O-αS groups by Sholl analysis. Two-way ANOVA followed by Sidak’s post-hoc test, n = 3. ∗∗∗p < 0.001. (F) Morphological (ending radius) changes in astrocytes in control, G2019S, O-αS, and G2019S+O-αS groups by Sholl analysis. Two-way ANOVA followed by Sidak’s post-hoc test, n = 3. ∗∗p < 0.01; ns, not significant. (G) Morphological (branching index) changes in microglia in control, G2019S, O-αS, and G2019S+O-αS groups by Sholl analysis. Two-way ANOVA followed by Sidak’s post-hoc test, n = 3. ∗p < 0.05; ns, not significant. (H) Morphological (ending radius) changes in microglia in control, G2019S, O-αS, and G2019S+O-αS groups by Sholl analysis. Two-way ANOVA followed by Sidak’s post-hoc test, n = 3. ∗p < 0.05; ns, not significant. Data are represented as mean ± SEM.

We then asked whether *LRRK2 G2019S* mutation promoted the effects of O-αS via regulating neuroinflammation. Immunostaining results showed that the number of astrocytes and microglia increased significantly after O-αS injection in both control and *LRRK2-G2019S*-Tg mice (Figure 2A). However, G2019S group showed more cell proliferation compared with the control group (Figures 2A–2C and S4). Morphological analysis showed that the cell ending radius and branch number of astrocytes significantly increased in the O-αS-treated G2019S group compared with the O-αS-treated control group (Figures 2D–2F), while the morphology of microglia did not change significantly (Figures 2D, 2G, and 2H). Our study revealed that astrocytic inflammation maybe pronounced at a certain stage during the development of PD.

### *LRRK2G2019S* enhanced O-αS-induced inflammatory levels in primary astrocytes, and inhibition of LRRK2 kinase activity could rescue the effects of both *LRRK2 G2019S* and O-αS

**Figure 3:**
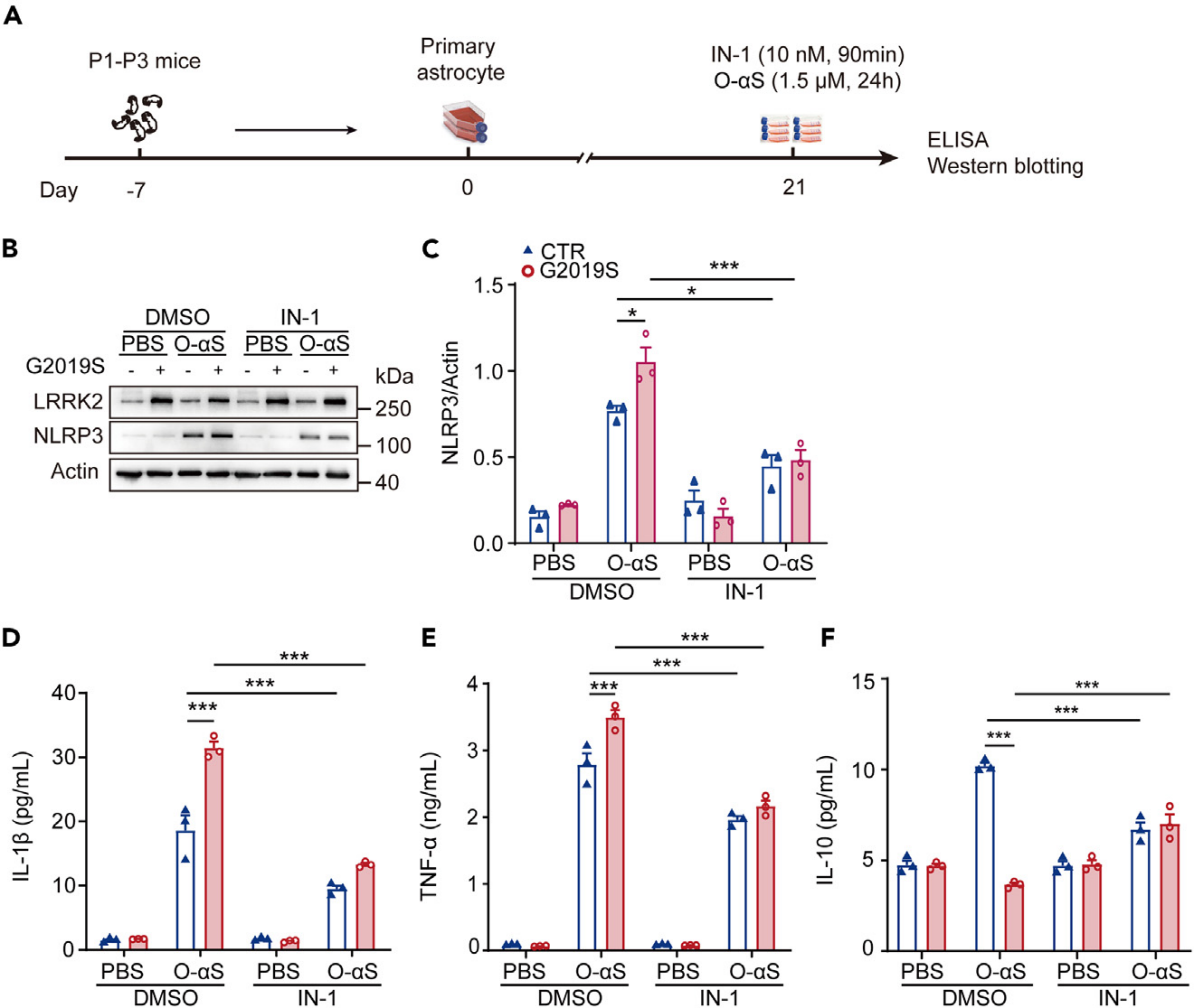
*LRRK2 G2019S* mutation exacerbated inflammatory response in O-αS-treated astrocytes, and IN-1 reduced the effects of *LRRK2 G2019S* and O-αS (A) Flow-process diagram of experimental treatment of primary astrocytes. (B and C) Western blotting assessment of NLRP3 protein expression in astrocytes in the control, G2019S, O-αS, and G2019S+O-αS groups. Two-way ANOVA followed by Sidak’s post-hoc test, n = 3. ∗p < 0.05; ∗∗p < 0.01; ∗∗∗p < 0.001; ns, not significant. (D‒F) The levels of IL-1β, IL-10, and TNF-α in supernatants of different treatments were determined by ELISA in the control, G2019S, O-αS, and G2019S+O-αS groups. Two-way ANOVA followed by Sidak’s post-hoc test, n = 3. ∗∗∗p < 0.001. Data are represented as mean ± SEM.

*In vivo* studies are difficult to explore the role of astrocyte-specific inflammatory responses in neuroinflammation, excluding the contribution of microglia. Both microglia and astrocytes were involved in the inflammatory responses. We also confirmed that NOD-like receptor thermal protein domain associated protein 3 (NLRP3) inflammasomes could co-localize with both microglia and astrocyte in O-αS-treated LRRK2 G2019S mouse (Figure S5). To resolve the difficulty, we used primary cultured astrocytes (labeling with GFAP) for further studies (Figure S6A). We also investigated whether expression of LRRK2 was time-dependent in primary astrocytes using western blotting. Expression of LRRK2 in primary astrocytes increased over time, and expression in the G2019S group was significantly higher than that in the control group after 21 days (Figures S6B and S6C). However, LRRK2 in primary microglia was barely detectable (Figure S6D). We cultured G2019S astrocytes for 21 days to study the effect of O-αS-induced neuroinflammation. Extracellular O-αS may interact with various cellular receptors, which can upregulate interleukin (IL)-1α, IL-1β, IL-6, and TNF-α transcription of proinflammatory factors.^16^ We treated cultured primary astrocytes with O-αS (0.5 μg/mL) and detected the inflammation-related factors 24 h later (Figure 3A). Western blotting results showed that O-αS induced cultured primary astrocytes to produce high levels of inflammation-associated NLRP3, and *LRRK2 G2019S* mutation significantly enhanced the effects of O-αS (Figures 3B and 3C). We collected the cell supernatant from each group and detected the inflammatory factors IL-1β and TNF-α and anti-inflammatory factor IL-10 using ELISA. Both IL-1β and TNF-α increased significantly in the G2019S+O-αS group (Figures 3D and 3E), while the level of IL-10 was significantly reduced in the G2019S+O-αS group (Figure 3F), compared with the CTR+O-αS group. We also utilized IN-1, an LRRK2 kinase inhibitor, to confirm the interplay between *LRRK2 G2019S* mutation and O-αS and found that IN-1 could significantly reduce the expression levels of NLRP3, IL-1β, and TNF-α in both O-αS group and G2019S+O-αS group (Figures 3B–3F), indicating that O-αS might act as inflammatory mediator via enhancing LRRK2 kinase activity.

### *LRRK2 G2019S* contributed to the inflammatory response through NF-κB pathway

**Figure 4:**
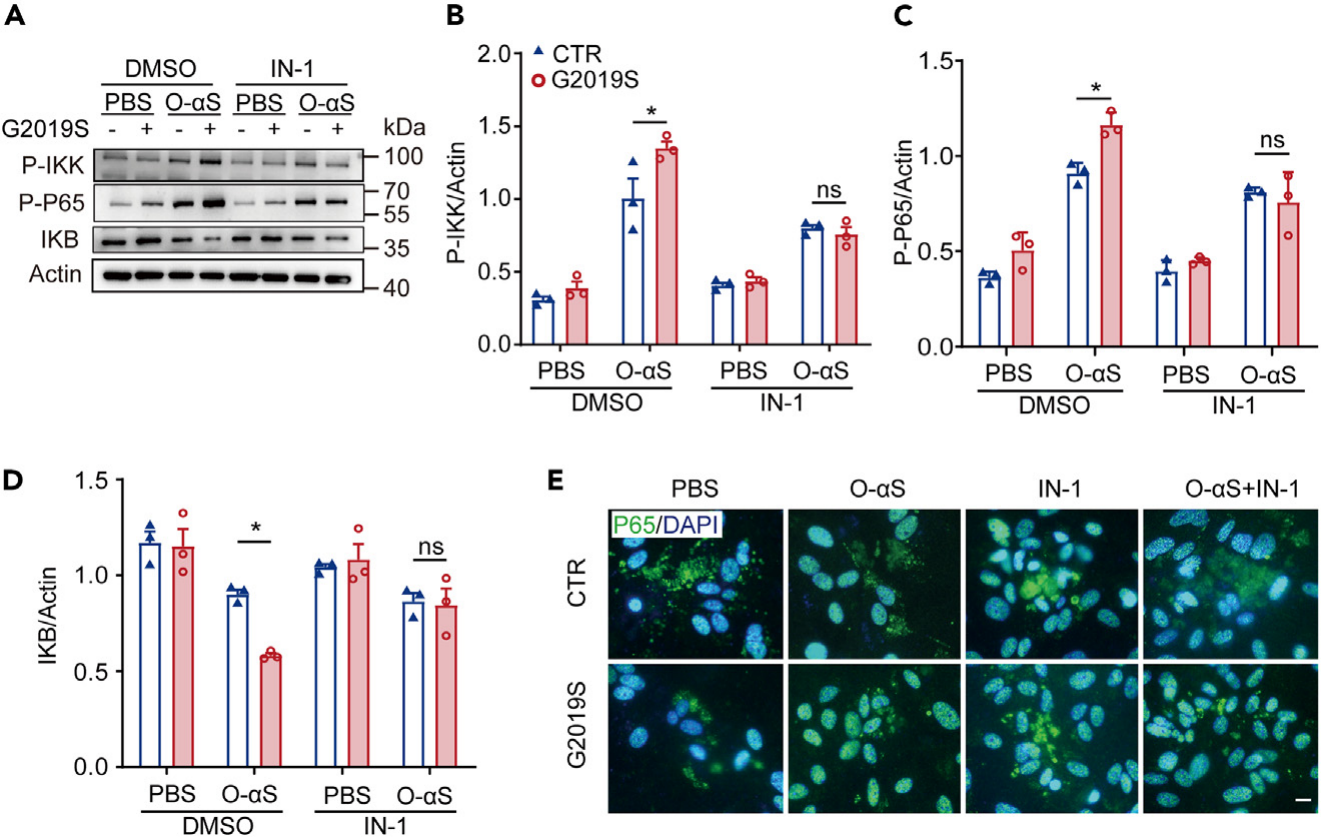
*LRRK2 G2019S* regulated inflammatory activity through NF-κB pathway (A) Western blotting assessment of P-IKK, P-P65, and IKB protein expression in astrocytes in the control, G2019S, O-αS, and G2019S+O-αS groups. (B‒D) Quantitative expression of P-IKK, P-P65, and IKB in astrocytes in the control, G2019S, O-αS, and G2019S+O-αS groups. Two-way ANOVA followed by Sidak’s post-hoc test, n = 3. ∗p < 0.05; ns, not significant. (E) Immunostaining for primary astrocyte on the substantia nigra of mice with anti-P65 antibody in the control, G2019S, O-αS, and G2019S+O-αS groups. Nuclei are stained with DAPI. (Scale bar = 50 μm， n = 3 per group). Data are represented as mean ± SEM.

NF-κB is an important transcription factor in the inflammatory pathway and mediates the pathological processes of neurodegeneration.^17^ To verify whether NF-κB was involved in the inflammation associated with *LRRK2 G2019S* mutation and O-αS, we detected the expression of phosphorylation-inhibitor of Kappa B Kinase (P-IKK), inhibitor kappa B alpha (IKB), and phosphorylation-p65 by western blotting. P-IKK and P-p65 were increased in the G2019S+O-αS group compared with O-αS group (Figures 4A–4C), and the level of IKB in the G2019S+O-αS group was significantly higher than that in O-αS group (Figures 4A and 4D). IN-1 reversed the changes in these protein levels affected by *LRRK2 G2019S* mutation (Figures 4A–4D). Immunofluorescence staining of p65 also revealed that *LRRK2 G2019S* mutation promoted the entry of p65 into the nuclei in both O-αS-treated and untreated groups, which could be blocked by IN-1 (Figure 4E). These results indicate that *LRRK2 G2019S* mutation might promote astrocytic neuroinflammation induced by O-αS via the NF-κB signaling pathway.

## Discussion

The penetrance of *LRRK2 G2019S* is incomplete, and thus it may be affected by the external environment or other genetic factors. The effects of *LRRK2 G2019S* are age dependent, and there was no loss of dopaminergic neurons in *LRRK2 G2019S*-Tg mice after a long-term observation.^18^ The present study showed that LRRK2 G2019S promoted dopaminergic cell loss and induced gait disturbance and motor learning deficit in O-αS-treated LRRK2 G2019S-Tg mice. However, no locomotion decline in G2019S+O-αS group was found in our study (data not shown). It is currently thought that classic motor symptoms of PD only become noticeable when the cell loss of dopaminergic neurons in SNpc reaches about 50% due to compensatory mechanisms. TH-positive cells were found to be decreased about 30% in G2019S+O-αS group compared with the control group, revealing that the animals presented early-stage parkinsonism at that time point. Meanwhile, no significant difference in TH-positive cells was found in the control+O-αS group compared with the control group. In A30P mice characterized by a rapid onset of α-syn pathology and the presence of O-αS across the whole brain, Anish et al. found that there was no loss of dopaminergic neurons, but dysregulation of the monoaminergic system was recorded in older mice (Behere et al., 2021). We speculate that the reduction of TH neurons induced by O-αS requires the combination of other disease-risk factors, such as aging, environmental effects, and PD-related variants.

Previous studies have shown that *LRRK2* mutations could promote the progression of PD and aggravate environmental toxin-induced inflammation.^19^^,^^20^^,^^21^ Meanwhile, knockout of LRRK2 gene prevents the neurotoxic effects and neuroinflammatory reactions caused by paraquat.^22^ Although these studies have revealed the improving effect of *LRRK2 G2019S* mutation in neurotoxin-induced neuroinflammation, it is still difficult to speculate how *LRRK2* is involved in the inflammatory processes. A clinical study has reported that there was a large concentration of O-αS in CSF of *LRRK2 G2019S* carriers.^9^ O-αS plays a crucial role in the pathological progression of PD, and *in vitro* experiments have shown that O-αS can be an inducing factor for neuroinflammation.^23^ The current study demonstrated that *LRRK2 G2019S* mutation exacerbates O-αS-induced inflammatory responses, and the effects could be rescued by LRRK2 kinase inhibitor. Although the numbers of microglia and astrocytes were significantly increased, only the morphology of astrocytes was found to be changed in the morphological analysis. Despite astrocytes taking up a great proportion of the brain cells, the contribution of astrogliosis to PD is still poorly understood. Although astrocytes perform a variety of physiological functions in the brain, they are pivotal mediators of α-syn toxicity since they internalize pathological α-syn released from damaged neurons.^24^ Astrocytes are considered to be instrumental amplifiers of neuroinflammation. Proinflammatory mediators such as IL-1β, TNF-α, and complement component 1q, as well as mitochondrial fragments released by activated microglia, can promote the activation of astrocytes to A1 proinflammatory state and lead to the further release of high levels of proinflammatory cytokines.^25^ Gabriel et al. proved that O-αS was rapidly engulfed by astrocytes and they were intracellularly stored, rather than degraded, resulting in impaired mitochondrial function.^26^ Meanwhile, oligomer-selective antibodies could prevent α-syn from accumulation and restore mitochondrial function in cultured astrocytes.^26^ These findings indicate that *LRRK2 G2019S* mainly increases the susceptibility to astrocytic neuroinflammation, leading to aggregation of NLRP3 and release of proinflammatory factors IL-1β and TNF-α.

The mice we used were *LRRK2 G2019S* systemic knockin, and *in vivo* research is difficult to explore the role of astrocyte-specific inflammatory response in neuroinflammation, excluding the role of microglia. Microglia are the primary immune cells in the brain, and microglial activation is correlated with PD severity.^27^ Several studies have reported LRRK2-mediated microglial inflammation in culture through NF-κB, NFATc2, and Toll-like receptor signaling pathways.^28^^,^^29^^,^^30^ However, other studies have demonstrated that the expression of LRRK2 in microglia is almost undetectable *in vivo* via numerous technical efforts.^31^^,^^32^^,^^33^ Microglia from adult wild-type mice and LRRK2-overexpressing mice also did not show any LRRK2 immunoreactivity.^34^ In LPS-induced inflammatory models, LRRK2 expression could only be detected in activated microglia after intracranial injection of LPS,^34^ while systemic administration of LPS did not induce microglial LRRK2 expression,^33^ revealing that the expressing of LRRK2 in microglia may require a strong inflammatory stress. In the present study, we found that *LRRK2 G2019S* promoted the activation of astrocytes after striatal injection of O-αS which is considered to be the most toxic strain leading to PD pathogenesis. Hence, we speculate that therapeutic efforts targeting astrocytic neuroinflammation might be an effective strategy to alleviate LRRK2-related PD.

Pathogenic protein aggregates, such as α-syn fibrils, another hallmark of PD pathology and the primary component of Lewy bodies, were reported to activate NLRP3 inflammasomes in microglia through interaction with Toll-like receptors and activation of NF-κB.^17^ To investigate the underlying mechanisms of the effects of *LRRK2 G2019S* on inflammation induced by O-αS, the NF-κB signaling pathway was studied. We demonstrated that O-αS significantly upregulated the expression of P-P65, P-IKK, and IKB (markers of NF-κB pathway activation). This is consistent with the previously reported results. More importantly, we found that *LRRK2 G2019S* mutation exacerbated the activation of NF-κB pathway and NLRP3 inflammasome. LRRK2 kinase inhibitor IN-1 significantly reduced the expression of NLRP3, pro-IL-1β induced by *LRRK2 G2019S*, and the levels of IL-1β and TNF-α. But what’s interesting is that inhibition of LRRK2 via IN-1 did not completely inhibit O-αS-mediated cytokine expression, which may support existence of LRRK2-independent inflammatory signaling pathway in PD-related neurodegeneration.

**Figure 5:**
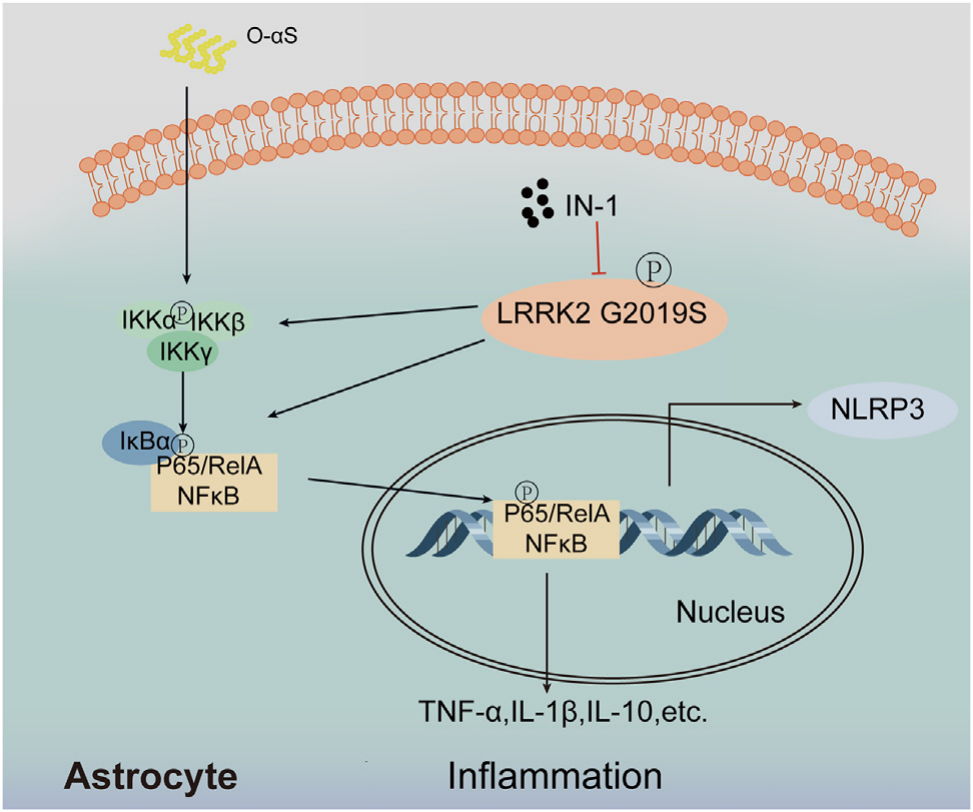
Schematic diagram for the mechanism of LRRK2 regulating inflammation in astrocytes induced by O-αS LRRK2 G2019S enhanced O-αS induced inflammatory levels in astrocytes through NF-κB pathway. Treatment with LRRK2 kinase inhibitor, IN-1, reduced NLRP3 inflammasome activation and the level of IL-1β and TNF-α and increased the level of IL-10.

In conclusion, our study verified that *LRRK2 G2019S*, cooperating with O-αS, altered the morphology of astrocytes, maintained inflammatory response, and eventually aggravated the loss of dopaminergic neurons. Treatment with LRRK2 kinase inhibitor, IN-1, reduced NLRP3 inflammasome activation and the level of IL-1β and TNF-α and increased the level of IL-10. We also demonstrated that *LRRK2 G2019S* participated in neuroinflammation via regulating the NF-κB pathway (Figure 5). Our results extended the current knowledge that *LRRK2 G2019S* acts as a promoting factor in O-αS-induced neuroinflammation.

### Limitations of the study

We confirmed the role of *LRRK2 G2019S* and O-αS in astrocytic inflammation. A key limitation of the study is that we could not exclude the effects of microglia on inflammatory responses *in vivo*. Furthermore, since the LRRK2 G2019S mouse we utilized has no region or cell type specific, it was difficult to confirm where O-αS actually acted on. Indeed, the loss of dopaminergic neurons should be a result of multiple factors, not neuroinflammation alone. It would be worthwhile to investigate the interplay of LRRK2 and O-αS in dopaminergic neurons.

## STAR★Methods

### Key resources table

**Table:** 

REAGENT or RESOURCE	SOURCE	IDENTIFIER
**Antibodies**		

Anti-TH	Sigma	T1299; RRID: AB_477560
Anti-GFAP	Abcam	ab7260; RRID: AB_305808
Anti-IBA1	Abcam	Ab5076; RRID: AB_2224402
Anti-LRRK2	Abcam	ab133474; RRID: AB_2713963
Anti-NLRP3	AdipoGen	AG-20B-0014-C100; RRID: AB_2885199
Anti-P-IKK	Cell Signaling Technology	2697S; RRID: AB_2079382
Anti-P-P65	Abcam	ab19870; RRID: AB_776753
Anti-IKB	Cell Signaling Technology	4812S; RRID: AB_10694416
Anti-α-syn	Abcam	Ab1903; RRID: AB_302665
Anti-β-actin	Sigma	A3854; RRID: AB_262011
Goat anti-mouse IgG-HRP	Jackson ImmunoResearch	115-001-003; RRID: AB_2338443
Goat anti-rabbit IgG-HRP	Jackson ImmunoResearch	111-001-003; RRID: AB_2337910
488-conjugated secondary antibody	Invitrogen	A11056; RRID: AB_2534103
594-conjugated secondary antibody	Invitrogen	A21429; RRID: AB_2535850

**Bacterial and virus strains**		

BL21 (DE3) competent cells	TransGen Biotech	CD601-02

**Chemicals, peptides, and recombinant proteins**		

Trypsin-EDTA (0.25%)	Gibco	25200072
Dulbecco’s modified Eagle’s medium/F12 Medium	Gibco	11330032
Penicillin and streptomycin	Gibco	15140122
Fetal bovine serum	Gibco	10099141C
Dulbecco’s modified Eagle’s medium	Gibco	11995065
DAPI	Vector Laboratories	H1200; RRID: AB_2336790
Isoflurane	RWD Life Science	R510-22
Phosphotungstic acid	Solarbio	G1870
H2-DCFDA	Invitrogen	D399
IN-1	MedChemExpress	HY-10875

**Critical commercial assays**		

HiTrap® Q FF column	GE Healthcare	17515601
Endotoxin removal spin columns	ThermoFisher Scientific	88276
Chromogenic LAL endotoxin assay kit	GenScript	L00350
DAB Kit	Genentech	GK500705
Mouse IL-1β Elisa kit	Thermo Fisher scientific	BMS6002
Mouse TNF-α Elisa kit	R&D Systems	DY410
Mouse IL-10 Elisa kit	R&D Systems	SM1000B

**Experimental models: Cell lines**		

PC12 cells	Chinese Academy of Sciences	TCR 9, RRID: CVCL_0481

**Experimental Models: Organisms/strains**		

Mouse: human LRRK2∗G2019S 2AMjff/J mice	Jackson Laboratory	Stock No: 018785; RRID: IMSR_JAX: 018785

**Recombinant DNA**		

pET21a-α-syn	Michael J. Fox Foundation	addgene plasmid: 51486

**Software and algorithms**		

ImageJ	NIH	RRID: SCR_003070
GraphPad Prism	GraphPad Software	RRID: SCR_002798
Illustrator CC	Adobe	RRID: SCR_010279

**Other**		

Tecnai G2 F20	FEI	Infinite 200Pro
Stoelting stereotaxic apparatus	RWD Life Science	68001
4–20% Mini-PROTEAN Precast Protein Gels	BIO-RAD	4561093

### Resource availability

#### Lead contact

Further information and requests for resources and reagents should be directed to and will be fulfilled by the Lead contact, Chun-Feng Liu (liuchunfeng@suda.edu.cn).

#### Materials availability

All unique/stable materials will be made available upon reasonable request from the lead contact without restriction.

### Experimental model and study participant details

This study does not involve any patients or healthy control participants.

#### Animals

LRRK2 transgenic mice with G2019S mutation (C57BL/6J-Tg LRRK2∗G2019S 2AMjff/J) were purchased from Jackson Laboratory (Bar Harbor, ME, USA) and hemizygous mice were bred to noncarrier (wild-type) siblings. And male mice aged 6 months were used throughout this study. Mice were raised in a relatively stable environment: light/dark ratio 12/12 h, temperature (21 ± 2°C), and relative humidity (55 ± 5%). Five animals were placed in one cage and had unrestricted access to food and water. All animal experiment protocols were approved by the Institutional Animal Care and Use Committee of Soochow University (Suzhou, China).

#### Cultured cells

Primary astrocytes were acquired from P1-P3 pups with identified genotypes. The cortex was isolated and dissociated in 0.1 M PBS, then digested with 0.25% trypsin (GIBCO) for 15 min, and centrifuged (1,000 rpm) after filtration with a 70-μm strainer. Primary astrocytes were resuspended and maintained in Dulbecco’s modified Eagle’s medium/F12 (Gibco, Grand Island, NY, USA) containing penicillin (100 U/mL) and streptomycin (100 U/mL), supplemented with 10% fetal bovine serum (Gibco, Grand Island, NY, USA). Microglia were depleted from the mixed culture through shaking at 220 rpm/min for 3 h. Astrocytes sub-cultured for 3∼6 generations were used in this study. LRRK2 kinase inhibitor IN-1 was purchased from MedChemExpress (HY-10875, Monmouth Junction, NJ, USA) and dissolved in DMSO.

PC12 cells were purchased from Institute of Cell Biology (Chinese Academy of Sciences, Shanghai, China) and cultured in Dulbecco’s modified Eagle’s medium (Gibco, Grand Island, NY, USA).

### Method details

#### Preparation of α-syn and generation of O-αS

The plasmid pET21a containing human α-syn cDNA (addgene plasmid 51486, Michael J. Fox Foundation, USA) was transformed into BL21 (DE3) *Escherichia coli* (TransGen Biotech, China). Protein expression was induced at OD_600_ 0.8 with 0.25 mM isopropyl-1-thio-D-galactopyranoside (IPTG) for 4 h at 37°C. The cell pellet was harvested and lysed using a tip sonicator in 100 mM Tris–HCl (pH 8.0) with 1 mM EDTA and 1 mM phenylmethylsulfonyl fluoride, and then boiled for 15 min. After centrifugation at 20,000 × g for 30 min, 20 mg/mL streptomycin was added to the supernatant to remove nucleic acids, followed by further centrifugation. Unwanted proteins were removed from the supernatant by adjusting pH to 3.5 using 1 M HCl. After centrifugation, the supernatant was dialyzed overnight against 20 mM Tris–HCl (pH 8.0). The sample was filtered with a 0.22-μm membrane (SLGP033RB, Millipore, USA) and purified by HiTrap® Q FF column (GE Healthcare, USA) and the purity of each peak fraction was analyzed by Coomassie blue stained SDS-PAGE. The protein concentration was quantified by measuring the absorbance at 280 nm using a NanoDrop2000 spectrophotometer (Thermo, USA). Endotoxin was cleaned using Pierce high-capacity endotoxin removal spin columns (88276, Thermo, USA) and measured to be less than 0.05 EU/mL using the ToxinSensor™ chromogenic LAL endotoxin assay kit (L00350, GenScript, USA). The purified protein was pooled, dialyzed with deionized distilled water, concentrated with a 3-kDa cutoff membrane (UFC900308, Millipore, USA), aliquoted and lyophilized. To generate β-sheet-rich O-αS, the protein was resuspended in PBS (pH 7.4) to a final concentration of 12 mg/mL, and statically incubated at 37°C for 22–24 h, followed by ultracentrifugation and filtration using a 100-kDa cutoff membrane (UFC910008, Millipore, USA), as previously described.^10^

#### Native-polyacrylamide gel electrophoresis (PAGE)

Coomassie blue-stained native-PAGE were performed using 4–20% Mini-PROTEAN Precast Protein Gels (4561093, BIO-RAD, USA) according to manufacturer instruction.

#### Reactive oxygen species (ROS) detection

PC12 cells were treated with α-syn monomer or O-αS (0.5 μg/mL) for 15 min and ROS levels were detected using H2-DCFDA (D399, Invitrogen, USA).

#### Transmission electron microscope (TEM) imaging

Protein samples were applied to carbon-coated copper grids and left to stand for 2 min followed by staining with 2% phosphotungstic acid (G1870, Solarbio, China) for 2 min. Micrographs were taken with a TEM (TECNAI G2 F20, FEI, USA) at 200 kV and 34,000 X magnification.

#### O-αS induced neuroinflammatory mouse model

Male mice aged 6 months were used throughout this study, consisting of *LRRK2 G2019S*-Tg and noncarrier (wild-type) from the cross above. The mice were anesthetized by 2% isoflurane inhalation and placed in a stoelting stereotaxic apparatus (68001, RWD Life Science, China). O-αS (1 μg/μL, 2 μL) was stereotactically injected into the right striatum according to the Elsevier brain atlas (compact 3rd edition) 0.5 mm anterior to the bregma, 2.0 mm to the midline, and 3.0 mm subdural. The same volume of PBS was injected into the left striatum as the control. The rate was kept at 0.2 μL/min and left for 10 min, then slowly retracted.

#### Immunofluorescence and immunohistochemical staining

Six weeks after treatment with O-αS, the mice were perfused with 4% paraformaldehyde. The mouse brains were then dehydrated with a series of 15% and 30% sucrose solutions at 4°C. Serial 20-μm-thick slices containing the midbrain were coronally cut with a freezing microtome (Leica, Wetzlar, Germany). For fluorescent staining, slices were blocked in 0.1% Triton X-100 with 5% bovine serum albumin (BSA) for 1 h and incubated with anti-IBA1 antibody and anti-GFAP antibody at 4°C overnight. After washing three times in PBS, and incubation with 594 (red) (A21429, 1:1000, Invitrogen, Carlsbad, CA, USA)-or 488 (green)-conjugated secondary antibody (A11056, 1:1000, Invitrogen) for 2 h. After three washes, the slices were stained with DAPI (H1200, Vector Laboratories, Burlingame, CA, USA) and visualization with a Zeiss microscope (AXIO SCOPE A1, Zeiss, Germany). For immunohistochemical staining, slices were blocked in 0.1% Triton X-100 with 10% BSA for 1 h and incubated with anti-Tyrosine hydroxylase (TH) (T1299, 1:1000, Sigma–Aldrich, St louis, MO, USA) at 4°C overnight. TH immunoreactivity was detected by DAB Kit (GK500705, Genentech, China). After dimethylbenzene permeabilization, the slices were sealed hermetically with neutral resin and visualized with a Zeiss microscope. Each mouse was selected at the same level of anatomical structure and TH^+^ neurons in 5 slides for each mouse were counted with Image J software by two blinded investigators.^35^^,^^36^ Fluorescence intensity of IBA1 and GFAP was analyzed using Fiji software (National Institutes of Health, Bethesda, MD, USA).

#### Gait performance

Gait performance was conducted using TreadScan gait analysis system (CleverSys Inc, Reston, VA, USA) as previously described.^37^ Briefly, each mouse was placed into the chamber with a treadmill. After training at 5 m/s speed for 2 min, the mouse was allowed to run freely at 17 m/s speed and a digitalized video (100 frames/sec) with 2,000 frames was recorded by high-speed camera below the walkway to collect the paw prints. The video was then analyzed using Treadscan 4.0 software (CleverSys Inc, Reston, VA, USA).

#### Motor learning test

Motor learning test was performed using Rota-rod training system as previously described with minor modifications.^38^ Before the test, the mouse was habituated to the Rota-rod apparatus (SA102, SANSBIO, Jiangsu, China) for 2 min at 4 rpm speed. Immediately, 3 trials each day (5 min, 1 hour interval) were carried out using an accelerating protocol from 4 to 60 rpm. The latency to fall off the apparatus was automatically recorded by the software. The experiment was performed for 3 days and the average of the three trials in each day was calculated for further analysis.

#### Western blotting

After treatment, primary astrocytes were lysed in RIPA lysis buffer (150 mmol/L NaCl, 25 mmol/L Tris–HCl, pH 7.6, 1% sodium deoxycholate, and 1% NP-40) with a phosphatase inhibitor and a protease inhibitor (Roche, Switzerland). Proteins were kept on ice and mixed with loading buffer. About 20–40 μg cell lysate was isolated by SDS-PAGE (8% or 12.5% gel) and transferred to a PVDF membrane (Millipore). PVDF membrane was blocked with 5% milk powder or 5% BSA in TBS containing 0.1% Tween-20 and incubated for 1 h at room temperature. Blots were incubated with the primary antibody at 4°C overnight. The primary antibodies used were: LRRK2 (ab133474, 1:8000, Abcam, Cambridge, UK), NLRP3 (AG-20B-0014-C100, 1:2000, AdipoGen, San Diego, CA, USA), P-IKK (2697S, 1:500, Cell Signaling Technology, Danvers, MA, USA), P-P65 (AB19870, 1:1000, Abcam, Cambridge, MA, USA), and IKB (4812S, 1:1000, Cell Signaling Technology) and incubated with the corresponding secondary antibody for 2 h. The proteins were visualized using ECL detection kits (Vazyme, China) with chemiluminescence (6000EXP, TOUCH, China).

#### Cytokine measurement

After treatment with O-αS, the cell supernatant was collected and centrifuged at 3,000 rpm for 5 min for detection. The concentration of interleukin (IL)-1β and TNF-α was determined by ELISA (Thermo Fisher Scientific, USA). IL-10 was quantified using ELISA from R&D Systems (Minneapolis, MN, USA).

#### Sholl analysis

We performed Sholl analysis of the morphology of microglia and astrocytes.^39^^,^^40^ In brief, images of mouse brain slices which immunostained with IBA1 or GFAP antibody were captured with OLYMPUS VS200. Using the Sholl Analysis plugin for automatic drawing analysis in Fiji software, immunoreactive areas in each region were thresholded, divided by area, and expressed in corresponding multiples. The ending radius and branching index were calculated for further analysis.

### Quantification and statistical analysis

All analyses were performed using GraphPad Prism 8 (GraphPad Software, La Jolla, CA, USA). Numerical data were compared with one-way ANOVA, two-way ANOVA or repeated measures ANOVA. All values are displayed as mean ± SEM. *P* < 0.05 was considered significant (∗*P* < 0.05, ∗∗*P* <0.01, ∗∗∗*P* < 0.001; ns, not significant).

## Data Availability

• All data reported in this paper will be shared by the lead contact upon request. • This paper does not report original code. • Any additional information required to reanalyze the data reported in this paper is available from the lead contact upon request.

## Supplemental information

Document S1. Figures S1‒S6 and Table S1
