# Supplementary material for: LRRK2 G2019S promotes astrocytic inflammation induced by oligomeric α-synuclein through NF-κB pathway
Source: iScience. 2023 Oct 5;26(11):108130. doi: 10.1016/j.isci.2023.108130 (PMC10590863; doi:10.1016/j.isci.2023.108130)
Supplement: Document S1. Figures S1‒S6 and Table S1 [file mmc1.pdf]

iScience, Volume *26*

Supplemental information

LRRK2 G2019S promotes astrocytic inflammation induced by oligomeric α-synuclein through NF-κB pathway

Kai-Jie He, Jin-Bao Zhang, Jun-Yi Liu, Feng-Lun Zhao, Xiao-Yu Yao, Yu-Ting Tang, Jin-Ru Zhang, Xiao-Yu Cheng, Li-Fang Hu, Fen Wang, and Chun-Feng Liu

Supplementary figures S1-S6 and table S1

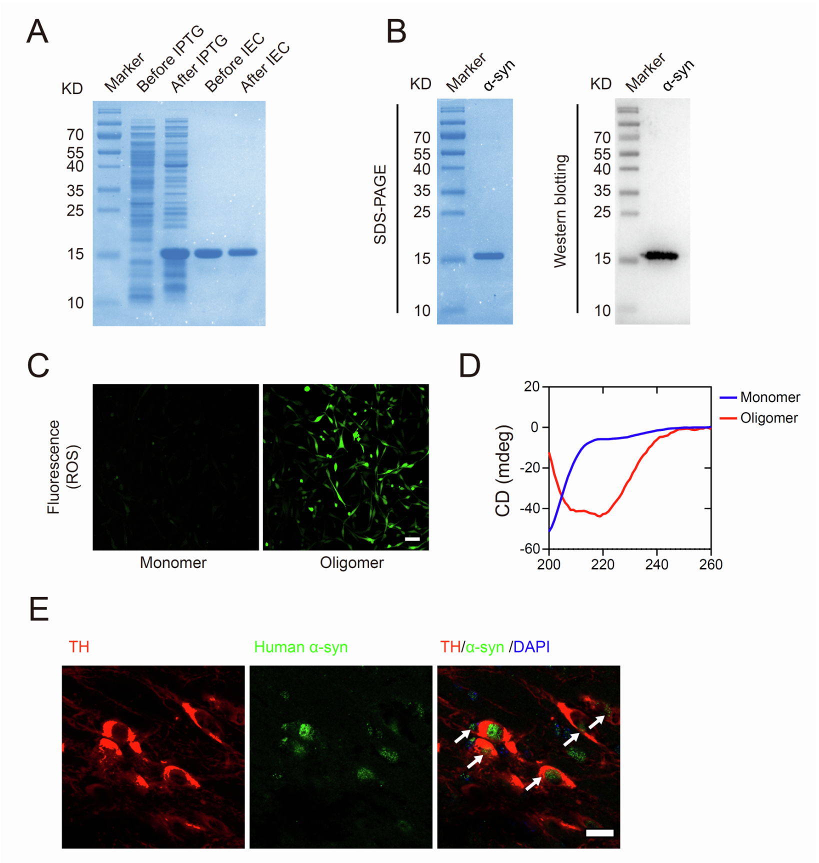

Figure S1. Preparation and identification of O-αS, related to Figure 1.

(A) Expression and purification of α-syn. Lane 1, protein molecular weight markers, labeled in kD; lane 2, uninduced total protein; lane 3, 4 h post-induction with IPTG; lane 4, crude purified total protein; lane 5, post- purified with IEC.

(B) Representative of α-syn identification by Coomassie Blue and Western blotting.

(C) ROS detection for identification of O-αS (scale bar=20 μm).

(D) CD spectra of the total α-syn monomer and oligomer.

(E) Co-localization of TH and human α-syn in SNc (scale bar=10 μm).

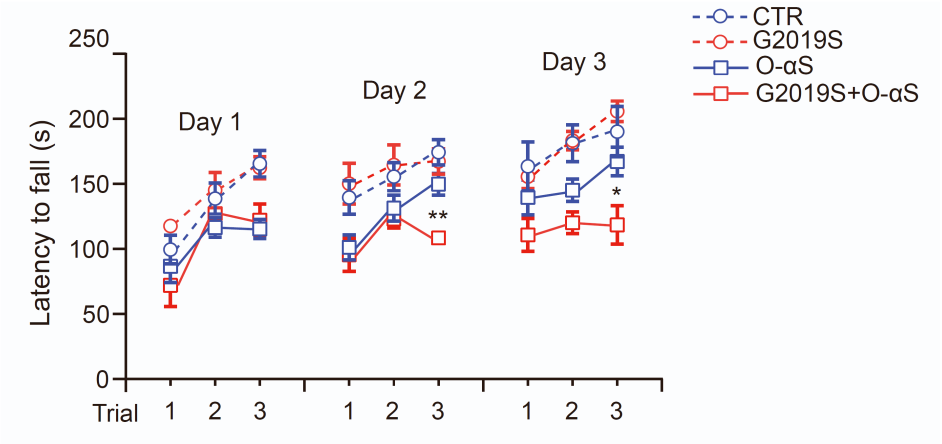

Figure S2. Rota-Rod data obtained from each date and trial, related to Figure 1.

Two-way ANOVA followed by Sidak's post-hoc test, n=3. *P < 0.05; **P < 0.01; ns, not significant. Data are represented as mean +/- SEM.

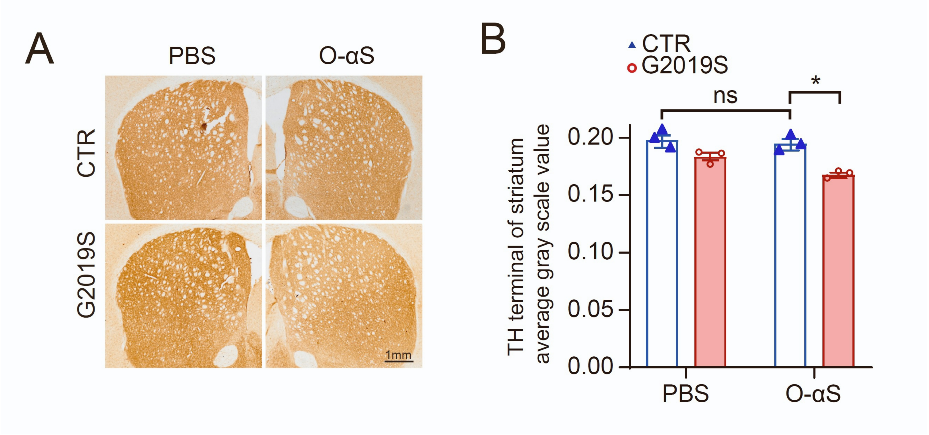

Figure S3. Representative images and quantification for TH terminal density in striatum, related to Figure 1.

(A) Representative images of IHC staining of TH terminal in the striatum of mice in the control, G2019S, O-αS and G2019S+O-αS groups. (scale bar 1 mm).

(B) Statistical comparison of TH terminal density in the striatum. Two-way ANOVA followed by Sidak's post-hoc test, n=3. *P < 0.05; ns, not significant. Data are represented as mean +/- SEM.

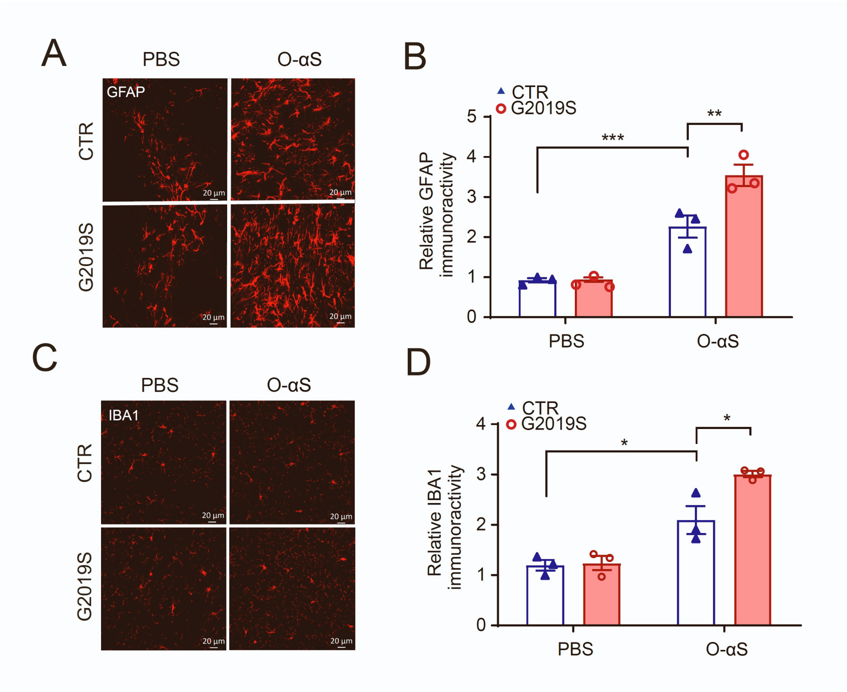

Figure S4. LRRK2 G2019S enhanced activation of glial cells in the striatum of O-αS-induced mouse model, related to Figure 2.

(A) Immunostaining for astrocyte in the striatum of mice with anti-GFAP antibody in the control, G2019S, O-αS and G2019S+O-αS groups. (Scale bar=20 μm).

(B) Statistical comparison of relative fluorescence intensity of control, G2019S, O-αS and G2019S+O-αS groups reflected activation of astrocytes. Two-way ANOVA followed by Sidak's post-hoc test, n=3. $**P < 0.01$; $***P < 0.001$. Data are represented as mean +/- SEM.

(C) Immunostaining for microglia in the striatum of mice with anti-IBA1 antibody in the control, G2019S, O-αS and G2019S+O-αS groups. (Scale bar 20 μm).

(D) Statistical comparison of relative fluorescence intensity of control, G2019S, O-αS and G2019S+O-αS groups reflected activation of microglia. Two-way ANOVA followed by Sidak's post-hoc test, n=3. $*P < 0.05$. Data are represented as mean +/- SEM.

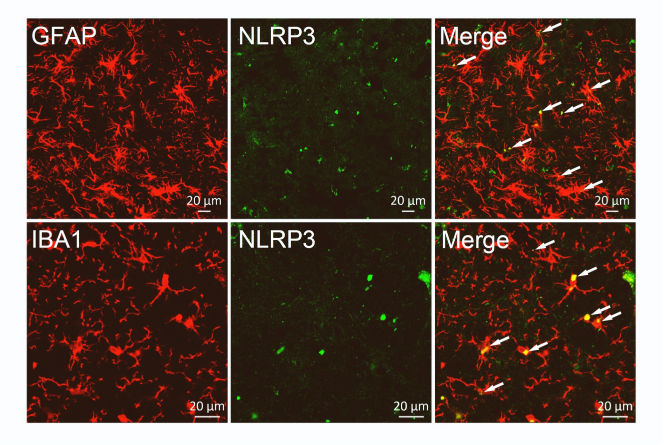

Figure S5. Co-localization of NLRP3 with GFAP or IBA1 in O-αS-treated LRRK2 G2019S mouse, related to Figure 2.

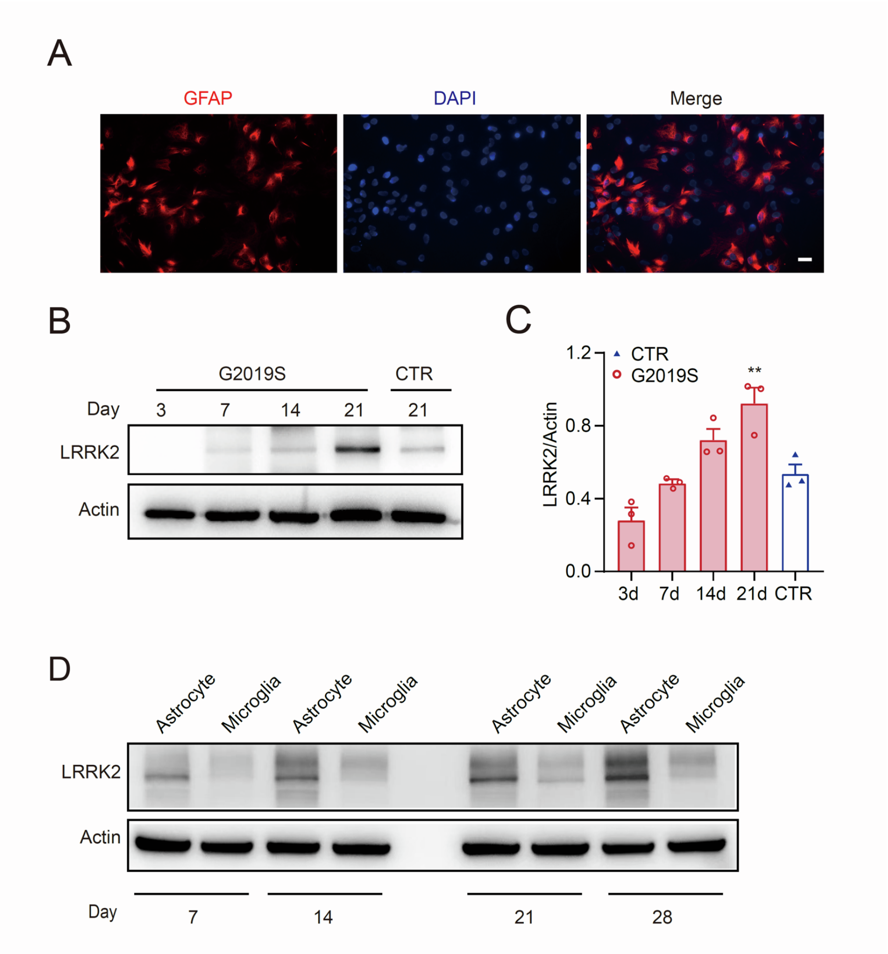

Figure S6. LRRK2 expression in primary astrocytes of CTR and G2019S mice, related to Figure 3 and Figure 4.

(A) Identification of primary astrocytes and microglia (scale bar 20.0 μm).

(B) Western blotting assessment of LRRK2 protein expression at 3, 7, 14 and 21 days in primary astrocytes in the control and G2019S mice.

(C) Statistical comparison of LRRK2 protein in primary astrocytes at different time points (3, 7, 14 and 21 days) was analyzed quantitatively. One-way ANOVA followed by Sidak's post-hoc test, n=3. **P < 0.01. Data are represented as mean +/- SEM.

(D) Western blotting assessment of LRRK2 protein expression in primary astrocytes and microglia in G2019S mice.

Table S1. Data for Rota-Rod experiment (Mean±SEM), related to Figure 1E.

Day1	CTR	G2019S	0- αS	G2019S+ 0- αS
Trail 1	99.4 ± 25.1 s	118.1 ± 8.1 s	86.5 ± 28.3 s	72.0 ± 36.3 s
Trail 2	138.6 ± 26.8 s	146.6 ± 30.5 s	116.4 ± 16.6 s	127.2 ± 24.8 s
Trail 3	165.6 ± 23.0 s	156.2 ± 19.2 s	115.2 ± 16.6 s	122.0 ± 28.1 s
Day2				
Trail 1	139.6 ± 29.0 s	141.6 ± 35.0 s	110.1 ± 21.7 s	95.5 ± 28.5 s
Trail 2	155.5 ± 24.1 s	163.6 ± 34.6 s	133.3 ± 22.4 s	123.1 ± 15.5 s
Trail 3	174.2 ± 22.0 s	167.9 ± 22.1 s	149.7 ± 18.7 s	108.5 ± 8.6 s
Day3				
Trail 1	163.5 ± 42.0 s	154.1 ± 20.7 s	139.4 ± 30.0 s	110.8 ± 28.7 s
Trail 2	181.1 ± 31.6 s	180.7 ± 16.1 s	145.1 ± 19.3 s	120.1 ± 18.7 s
Trail 3	190.1 ± 43.6 s	206.6 ± 17.9 s	167.1 ± 24.8 s	118.4 ± 32.9 s
